# Medical Morphology Training Using the Xuexi Tong Platform During the COVID-19 Pandemic: Development and Validation of a Web-Based Teaching Approach

**DOI:** 10.2196/24497

**Published:** 2021-03-15

**Authors:** Qinlai Liu, Wenping Sun, Changqing Du, Leiying Yang, Na Yuan, Haiqing Cui, Wengang Song, Li Ge

**Affiliations:** 1 Department of Pathology Shandong First Medical University & Shandong Academy of Medical Sciences Tai’an China; 2 Department of Histology and Embryology Shandong First Medical University & Shandong Academy of Medical Sciences Tai’an China; 3 Department of Immunology Shandong First Medical University & Shandong Academy of Medical Sciences Tai’an China

**Keywords:** COVID-19, histology and embryology, pathology, web-based teaching, Xuexi Tong platform

## Abstract

**Background:**

Histology and Embryology and Pathology are two important basic medical morphology courses for studying human histological structures under healthy and pathological conditions, respectively. There is a natural succession between the two courses. At the beginning of 2020, the COVID-19 pandemic suddenly swept the world. During this unusual period, to ensure that medical students would understand and master basic medical knowledge and to lay a solid foundation for future medical bridge courses and professional courses, a web-based medical morphology teaching team, mainly including teachers of courses in Histology and Embryology and Pathology, was established.

**Objective:**

This study aimed to explore a new teaching mode of Histology and Embryology and Pathology courses during the COVID-19 pandemic and to illustrate its feasibility and acceptability.

**Methods:**

From March to July 2020, our team selected clinical medicine undergraduate students who started their studies in 2018 and 2019 as recipients of web-based teaching. Meanwhile, nursing undergraduate students who started their studies in 2019 and 2020 were selected for traditional offline teaching as the control group. For the web-based teaching, our team used the Xuexi Tong platform as the major platform to realize a new “seven-in-one” teaching method (ie, videos, materials, chapter tests, interactions, homework, live broadcasts, and case analysis/discussion). This new teaching mode involved diverse web-based teaching methods and contents, including flipped classroom, screen-to-screen experimental teaching, a drawing competition, and a writing activity on the theme of “What I Know About COVID-19.” When the teaching was about to end, a questionnaire was administered to obtain feedback regarding the teaching performance. In the meantime, the final written pathology examination results of the web-based teaching and traditional offline teaching groups were compared to examine the mastery of knowledge of the students.

**Results:**

Using the Xuexi Tong platform as the major platform to conduct “seven-in-one” teaching is feasible and acceptable. With regard to the teaching performance of this new web-based teaching mode, students demonstrated a high degree of satisfaction, and the questionnaire showed that 71.3% or more of the students in different groups reported a greater degree of satisfaction or being very satisfied. In fact, more students achieved high scores (90-100) in the web-based learning group than in the offline learning control group (*P*=.02). Especially, the number of students with objective scores >60 in the web-based learning group was greater than that in the offline learning control group (*P*=.045).

**Conclusions:**

This study showed that the web-based teaching mode was not inferior to the traditional offline teaching mode for medical morphology courses, proving the feasibility and acceptability of web-based teaching during the COVID-19 pandemic. Our findings lay a solid theoretical foundation for follow-up studies of medical students.

## Introduction

The outbreak of COVID-19 rapidly became a worldwide pandemic [1] and necessitated rapid changes to higher education worldwide [2]. Many educators were dedicated to deliver knowledge via distance learning and web-based pedagogies, without stopping the teaching process [3]. This is an unprecedented form of teaching, and it has created substantial challenges to teaching as well as unprecedented opportunities. The Ministry of Education in China called for “suspension of classes without suspending the school” [4]. With active response to the call of the country, the teaching team, mainly including teachers of histology and embryology and pathology courses, immediately organized and strived to explore the web-based teaching mode for medical morphology. In this new era of information, web-based learning creates conditions for life-long learning and greater flexibility for people to learn on their own personal time; also, varied locations, good availability, and cost-effectiveness are associated with web-based teaching [5-9]. However, web-based teaching still has the disadvantage of learners feeling isolated within the virtual environment [10]. To ensure the effectiveness of teaching, diverse teaching practices that improve the communication between teachers and students during web-based education have been conducted, promoting the autonomy of students in learning.

Histology and Embryology is a medical morphological foundation course that studies the microstructure of the healthy body, organ functioning, and embryonic development using a microscope [11]. Additionally, Pathology is a course on the histological structural changes of disease conditions in the body; it explores the etiology as well as the pathogenesis of disease, functional changes, and basic outcomes [12]. Pathology is a bridge course that is based on the Histology and Embryology course, and it is included in basic medicine and clinical medicine courses.

Both these courses are theoretical and practical basic courses, and previous theoretical and experimental teaching and assessment models were no longer considered to be suitable during the global situation of the COVID-19 pandemic. Therefore, it is necessary to adjust the teaching and training strategies for medical students to ensure successful completion of the curriculum [13]. To enable students to smoothly transition from the study of histology and embryology to the study of pathology, teachers from both departments should cooperate closely and create new teaching modes.

Web-based teaching has placed greater demands on teachers’ own qualities. To transform traditional classroom teaching to web-based teaching, teachers must master web-based teaching software and lead students to use virtual experiment platforms to grasp the essence of some morphological tissue sections. Therefore, teachers should consider students as the main body while the teachers themselves play the leading role, as in, “teach by learning and research by teaching.” In this study, our team teaches Histology and Embryology and Pathology courses as points of entry to explore a new medical morphological web-based teaching mode and methodology using the Xuexi Tong platform as the major approach. This provides a reference sample for teaching at the Shandong First Medical University and other associated medical universities during the epidemic and postepidemic eras.

## Methods

### Participants

This study was conducted in the Shandong First Medical University from March to July 2020. Although all clinical medicine undergraduate students in who began their studies in 2018 and 2019 (Grades 2018 and 2019) have applied the Xuexi Tong platform throughout their studies, 512 students from 10 teaching classes were selected as the web-based teaching experiment group, which included 254 students learning histology and embryology in Grade 2019 (freshmen) and 258 students learning pathology in Grade 2018 (sophomores). Among these 512 students, 253 (49.4%) were from rural areas, 259 (50.6%) were from urban areas, 508 (99.2%) were Han Chinese, and 4 (0.8%) were members of ethnic minority groups (2 Hui, 1 Manchu, and 1 Mongol, respectively). At present, as the COVID-19 pandemic has been basically brought under control in China, most students have returned to school. For comparison with the performance of traditional offline teaching, 5 classes of Grade 2020 (freshmen, nursing majors) were used as the control group for the Histology and Embryology course, and 5 classes of Grade 2019 (sophomores, nursing majors) were used as the offline teaching control group of the Pathology course. In the meantime, to demonstrate differences in the written test scores, the final written examinations in Pathology of Grade 2018 (web-based teaching) and Grade 2017 (traditional offline teaching) students were compared among different score intervals.

### Design of Teaching Methods

All the selected web-based teaching students did come into contact with other web-based teaching platforms, such as the Zhihui Shu platform, in other courses; however, we guaranteed that they had not come into contact with the Xuexi Tong platform before participating in this study.

Teaching standards were followed according to the normal teaching time, teaching content, and plans; also, attention was paid to attendance, homework, teaching content, and assessment.

#### Before Class

The electronic textbook and PowerPoint presentations were uploaded to the Xuexi Tong platform for students to preview and download. Content from Chinese University Massive Open Online Course (MOOC) and the school MOOC for the class was selected, and students were instructed to proceed to the related platform to learn.

#### Classroom

Sign-in 10 minutes before class was recommended. The important and difficult points of the courses were recorded as videos by the teacher and uploaded in the task point section of the Xuexi Tong platform. Students were informed of the classroom content and time schedule in advance, and the classroom tests and quick response questions were distributed at regular intervals. Before the end of the class, a certain amount of time was set for problem-solving, and teachers were expected to provide timely answers in the web-based chat group. After each chapter, a case discussion question related to the teaching content was arranged in the discussion area of the Xuexi Tong platform, and the teachers provided scores and comments based on the students’ analyses.

#### After Class

Once per week, a Tencent Meeting live broadcast question and answer link was sent to the students to check the deficiencies in web-based teaching knowledge and to address the problems encountered by the students. Teachers were asked to use EV screen recording software to record the important and difficult content of the chapter and upload the videos to the Xuexi Tong platform for students to review and summarize as well as to guide them in writing the chapter mind maps.

During the COVID-19 epidemic, to more objectively evaluate the learning effects of students under this new teaching mode, the final grade was divided into two parts. The final written grade accounted for 60% of the grade, and the formative evaluation accounted for 40%. The formative evaluation also had two parts; the writing of experimental reports accounted for 15% of the grade, and web-based learning accounted for 25%. The web-based learning included sign-in (10%), case analysis (10%), chapter tests (10%), task point viewing (30%), classroom tests (10%), interaction (5%), flipped classroom performance (10%), theme activity performance (5%), and mind map writing (10%).

### Applying the Flipped Classroom Teaching Mode

In the “Respiratory System” chapter, the team used Tencent Live Conference to “flip the classroom.” The teachers extracted 10 concepts from the “Respiratory System” chapter: (1) anatomical structure, microstructure, and embryogenesis of the respiratory system, (2) lobar pneumonia, (3) lobar pneumonia, (4) interstitial pneumonia, (5) chronic bronchitis, (6) chronic obstructive pulmonary emphysema, (7) silicosis, (8) pulmonary heart disease, (9) lung cancer, and (10) nasopharyngeal carcinoma.

Before the class, the students were asked to form study groups using WeChat and QQ to flexibly learn using various resources. The students learned the contents of the MOOC and task points, and they were divided into 10 random groups based on the concept of the group's presentation. Each group chose a representative to speak and give the presentation through Tencent Live Conference (Multimedia Appendix 1). At the end of the class, the teacher provided comments, and every student could go to the Xuexi Tong platform to rate the expression of the topic. The maximum total score of the flipped classroom presentation for each group was 10, and it was divided into three parts; the score within the group ratings accounted for 20%, the between-group ratings accounted for 40%, and the teacher’s rating accounted for 40%.

### Screen-to-Screen Experimental Teaching

Our team selected a medical morphological digital teaching platform [14] to guide students in observing virtual sections. The teacher carefully selected the most suitable tissue sections, and they used EV screen recording software to make videos to provide a dynamic view of the tissue sections and the PowerPoint presentations of the lectures. The videos were uploaded to the group chat section of the Xuexi Tong platform during the classes, and each student was required to post the picture they had drawn to the group chat; later, the teacher provided comments on the drawings.

### Organizing a Drawing Competition

Students were encouraged to actively participate in the “Morphological Drawing Competition of College Students in Medical Colleges” organized by the Basic Medicine Group of the Joint Association of the National Experimental Teaching Demonstration Center of Colleges and Universities. The drawings that required evaluation were divided into two groups: the Histology and Embryology drawing group and the Pathology drawing group. The Pathology drawing group was then divided into two subgroups, namely Gross Pathology and Histopathology. Finally, 10 drawings were selected to proceed further and were then sent to the organizing committee as entries in the competition.

### Conducting a Theme Activity

Before conducting a theme activity, the teachers sent many links on COVID-19 to students through WeChat in advance to enrich their knowledge. The topic of this theme activity was “What I Know About COVID-19.” The answer format of the topic was not limited, and the submission could be an essay, poem, song, or even a video based on the medical knowledge the student had learned.

### Evaluation and Statistical Analysis

Two weeks before the end of the teaching, a Questionnaire Star survey was used to investigate the long-term application performance of the new web-based teaching model and the traditional offline teaching model. The designed questions were imported into Questionnaire Star, and a WeChat link was generated and sent to the students. Next, the students answered the questions directly via the WeChat interface. Participation was voluntary, and complete anonymity was ensured. After conducting the survey, the final statistical results were automatically obtained and analyzed through Questionnaire Star.

In addition, as the Grade 2018 clinical undergraduates returned to the university at the end of August 2020, they underwent a written examination on pathology in their classroom. Therefore, SPSS version 19.0 (IBM Corporation) was used to compare the written examination results of these students with those of the clinical undergraduates of the five classes of Grade 2017 who were taught offline the previous year. The corresponding classes of the two grades were taught by the same teacher, and the question type of the final written examination retained the same difficulty, and *P*<.05 was considered to be statistically significant. GraphPad Prism (GraphPad Software) was used to generate the corresponding histograms.

## Results

### The New Web-Based Teaching Model Achieves Good Teaching Performance

**Figure 1:**
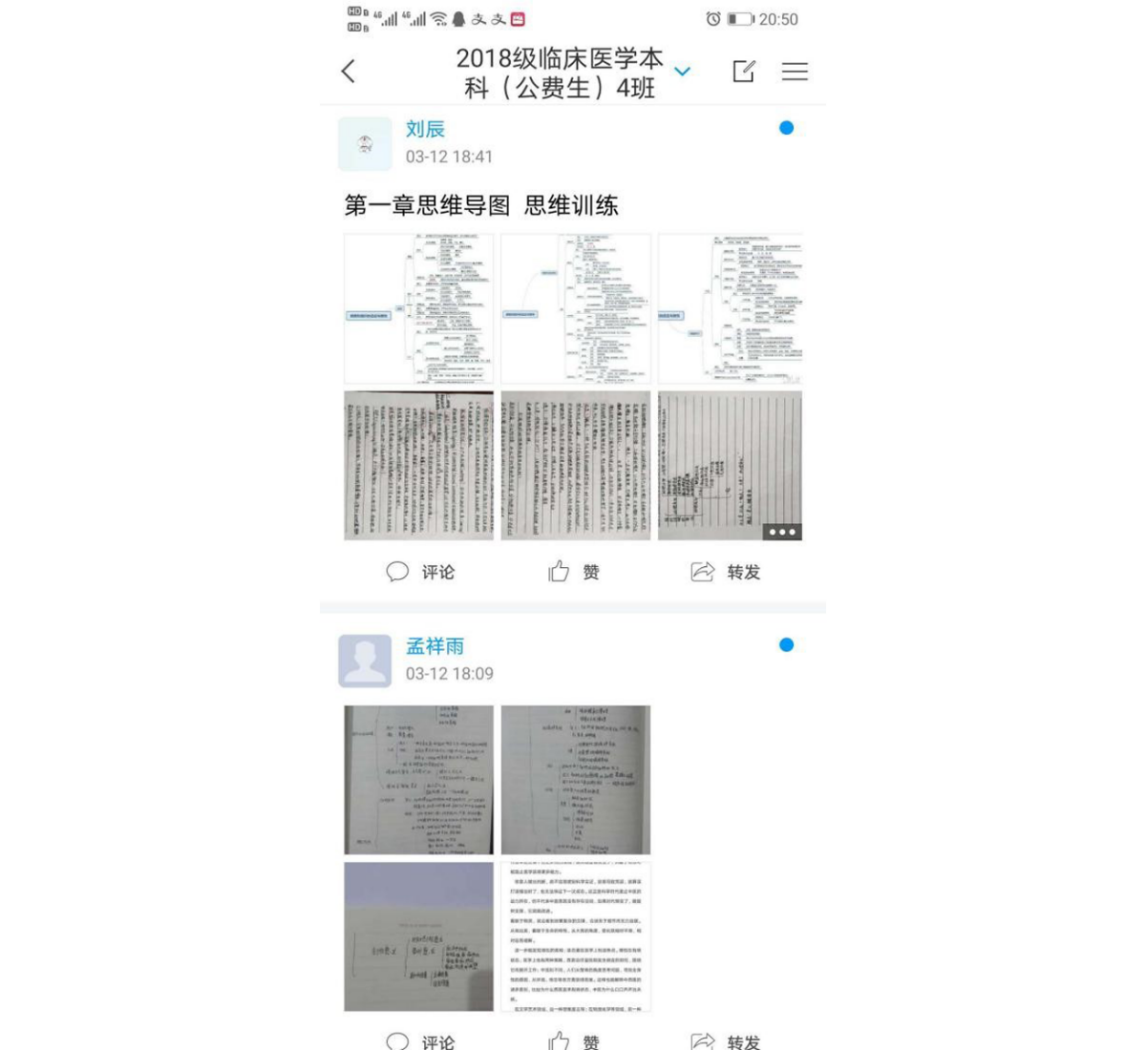
The mind map exercise performed by the students.

All the students completed the task points on time. Most of the students took the initiative to discuss case analysis, and they submitted their writings with regard to the mind map on time (Figure 1). Additionally, most of the students scored above 90 in the web-based learning evaluation.

All groups scored 9 or above on the flipped classroom assignment. This activity increased the students’ interest in learning and enhanced their learning and collaboration abilities.

During screen-to-screen experimental teaching, based on the teachers’ comments, most of the students mastered the content of the teaching task during the classroom, drew ideal pictures as required by the teacher, and posted their pictures in the group chat.

**Figure 2:**
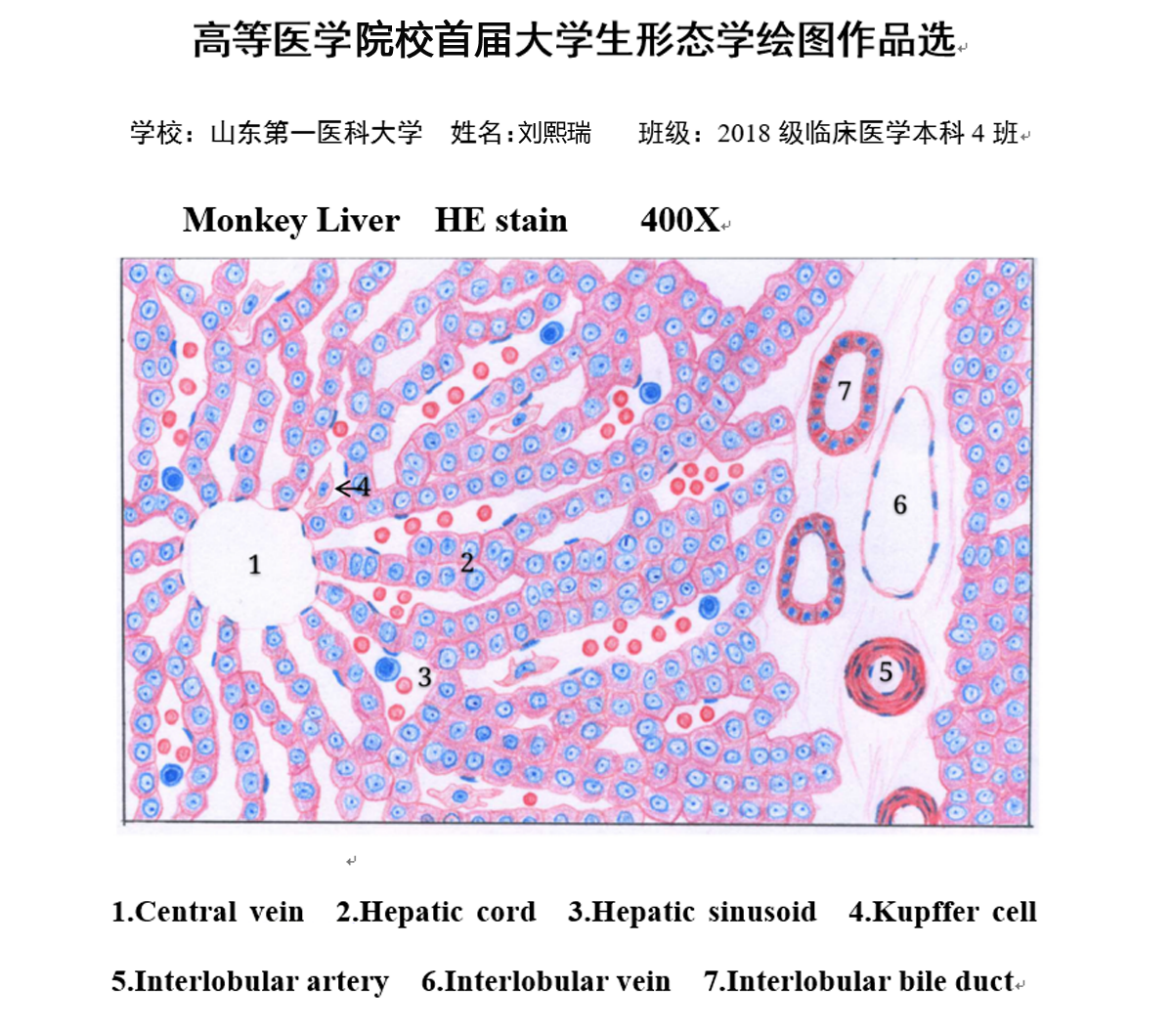
The winning entry in the drawing competition of the Histology and Embryology course.

**Figure 3:**
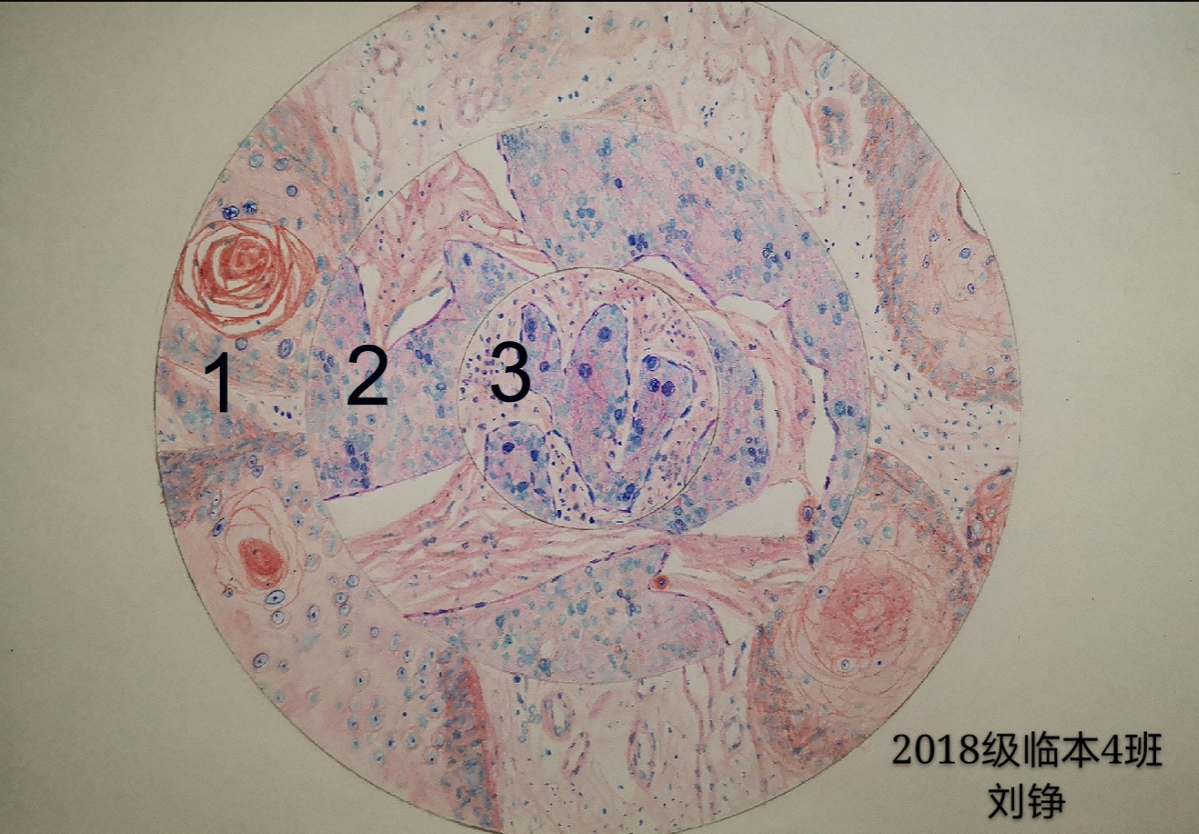
Representative image of pathological sections at low (1), medium (2) and high resolution power (3).

When participating in the drawing competition, all students drew good pictures: some of them used computer drawing software, while a few drew by hand. The subjects of the students’ drawings included a general specimen of lung cancer, the normal structure of liver tissue (Figure 2), the normal structure of the small intestinal villi, squamous cell carcinoma (Figure 3), chronic pulmonary congestion, pneumonia caused by COVID-19, and fatty liver. After the teacher’s instructions, all the participants drew better and more accurately.

**Figure 4:**
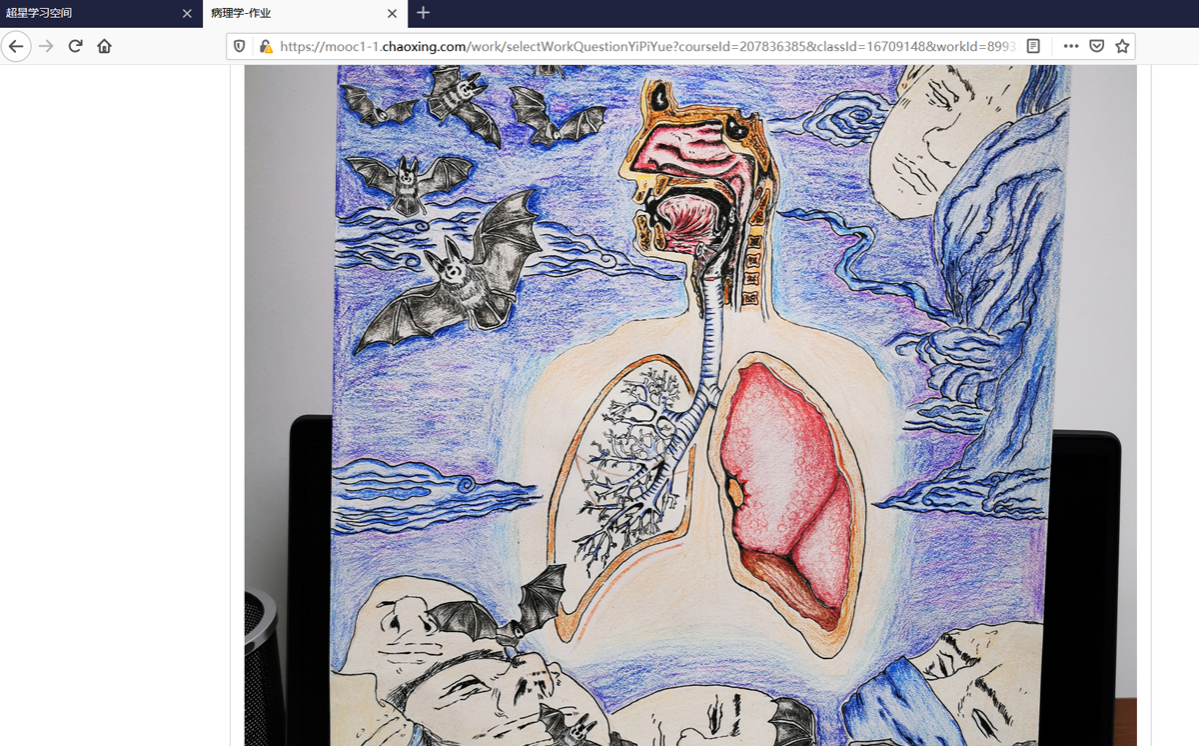
Representative drawing by a student for the theme activity.

Based on the theme activity, the students not only mastered the professional knowledge but also expressed their values. The students responded enthusiastically to the activity, and each student uploaded an assignment through the Xuexi Tong platform; responses included long papers and references, good poems, adapted song lyrics, meaningful pictures (Figure 4), PowerPoint presentations, and recordings of uplifting videos.

### Long-term Evaluation

Through the semester of web-based teaching practice, the results of the questionnaire survey and final written examination indicated that the new web-based teaching model was not inferior to the traditional teaching model. A total of 244 valid Histology and Embryology questionnaires and 246 valid Pathology questionnaires were answered by the students in the web-based teaching experiment group. The response rate of the students to the questionnaire was 95.7% (490/512). The rates of students who reported being very satisfied or having a greater degree of satisfaction with the theoretical teaching mode were 71.3% (174/244) in the Histology and Embryology course and 82.5% (203/246) in the Pathology course (Table 1). In terms of feelings about using the experimental platform, the rates of students who were very satisfied or had aa greater degree of satisfaction were 70.9% (173/244) in the Histology and Embryology course and 80.1% (197/246) in the Pathology course (Table 1), respectively. As shown in Table 2, considering the effectiveness of web-based teaching of Histology and Embryology and Pathology courses, more than 18.4% and 23.2% of the students in the experimental groups thought that the web-based courses were better than traditional teaching. Moreover, in the control group, a total of 253 valid Histology and Embryology questionnaires and 250 valid Pathology questionnaires were received through the Questionnaire Star tool. The effective ratio was 97.5% (503/516). As shown in Table 3, more than 7.1% (18/253) of students thought that the offline learning effect of the Histology and Embryology course was inferior to the web-based learning effect, while more than 11.6% (29/250) thought that the offline learning effect of the Pathology course was inferior to the web-based learning effect.

**Table 1:** Results of the first part of the questionnaire showing the students’ satisfaction with the web-based teaching.

Survey question and course		Responses, n (%)^a^				
		Very satisfied	A greater degree of satisfaction	Generally satisfied	Less satisfied	Dissatisfied
**1. What do you think of the current teaching mode? Are you satisfied?**						
	Histology and Embryology	64 (26.2)	110 (45.1)	57 (23.4)	7 (2.9)	6 (2.5)
	Pathology	78 (31.7)	125 (50.8)	36 (14.6)	3 (1.2)	4 (1.6)
**2. Is the web-based platform satisfactory?**						
	Histology and Embryology	63 (25.8)	110 (45.1)	58 (23.8)	8 (3.3)	5 (2.0)
	Pathology	85 (34.6)	112 (45.5)	36 (14.6)	8 (3.3)	5 (2.0)
**3. Do you think the current teaching goals are clear?**						
	Histology and Embryology	73 (29.9)	109 (44.7)	51 (20.9)	7 (2.9)	4 (1.6)
	Pathology	92 (37.4)	113 (45.9)	36 (14.6)	3 (1.2)	2 (0.8)
**4. Do you think the current teaching arrangements are reasonable?**						
	Histology and Embryology	71 (29.1)	114 (46.7)	50 (20.5)	7 (2.9)	2 (0.8)
	Pathology	102 (41.5)	116 (47.2)	24 (9.8)	2 (0.8)	2 (0.8)
**5. What is your attitude toward the quality of the web-based teaching microvideos?**						
	Histology and Embryology	53 (21.7)	114 (46.7)	67 (27.5)	7 (2.9)	3 (1.2)
	Pathology	88 (35.8)	111 (45.1)	37 (15.0)	7 (2.9)	3 (1.2)

^a^Percentages calculated based on 244 valid Histology and Embryology questionnaires and 246 valid Pathology questionnaires.

**Table 2:** Results of the second part of the questionnaire showing the students’ satisfaction with the web-based teaching performance.

Survey content and course		Responses, n (%)^a^			
		Very helpful, better than traditional classroom teaching	Helpful, similar to traditional classroom teaching	Helpful, but not as good as traditional classroom teaching	Not helpful
**1. In terms of improving learning efficiency and quality and solving difficult problems**					
	Histology and Embryology	47 (19.3)	96 (39.3)	95 (38.9)	6 (2.5)
	Pathology	57 (23.2)	97 (39.4)	88 (35.8)	4 (1.6)
**2. In terms of mobilizing students’ learning enthusiasm and initiative**					
	Histology and Embryology	48 (19.7)	93(38.1)/	94 (38.5)	9 (3.7)
	Pathology	58 (23.6)	90(36.6)	89 (36.2)	9 (3.7)
**3. In terms of knowledge summary, integration, application, and systematic understanding and memory**					
	Histology and Embryology	45 (18.4)	97 (39.8)	94 (38.5)	8 (3.3)
	Pathology	57 (23.1)	100 (40.7)	84 (34.1)	5 (2.0)
**4. For unity and cooperation, language and communication skills, and problem-solving**					
	Histology and Embryology	46 (18.9)	96 (39.3)	92 (37.7)	10 (4.1)
	Pathology	61 (24.8)	92 (37.4)	84 (34.1)	9 (3.7)

^a^Percentages calculated based on 244 valid Histology and Embryology questionnaires and 246 valid Pathology questionnaires.

**Table 3:** Results of the survey questionnaire showing the students’ satisfaction with the offline teaching performance in the control group.

Survey item and course		Responses, n (%)^a^			
		Very helpful, better than web-based classroom teaching	Helpful, similar to web-based classroom teaching	Helpful, but inferior to web-based classroom teaching	Not helpful
**1. In terms of improving learning efficiency and quality and solving difficult problems**					
	Histology and Embryology	118 (46.6)	105 (41.5)	25 (9.9)	5 (2.0)
	Pathology	115 (46)	95 (38.0)	39 (15.6)	1 (0.4)
**2. In terms of mobilizing students’ learning enthusiasm and initiative**					
	Histology and Embryology	129 (51.0)	99 (39.1)	20 (7.9)	5 (2.0)
	Pathology	121 (48.4)	93 (37.2)	35 (14.0)	1 (0.4)
**3. In terms of knowledge summary, integration, application, and systematic understanding and memory**					
	Histology and Embryology	123 (48.6)	95 (37.5)	27 (10.7)	8 (3.2)
	Pathology	115 (46.0)	92 (36.8)	38 (15.2)	5 (2.0)
**4. For unity and cooperation, language and communication skills, problem-solving**					
	Histology and Embryology	138 (54.5)	89 (35.2)	18 (7.1)	8 (3.2)
	Pathology	131 (52.4)	89 (35.6)	29 (11.6)	1 (0.4)

^a^Percentages calculated based on 253 valid Histology and Embryology questionnaires and 250 valid Pathology questionnaires.

**Figure 5:**
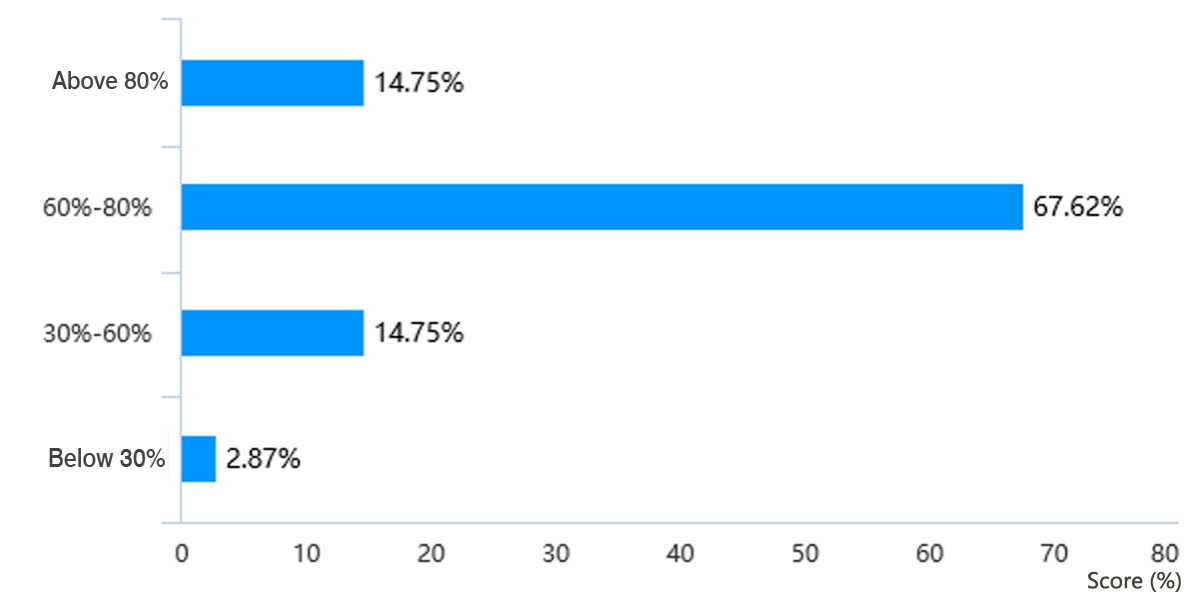
Scores showing the extent of students’ understanding and mastery of the theoretical knowledge of histology and embryology through web-based learning.

**Figure 6:**
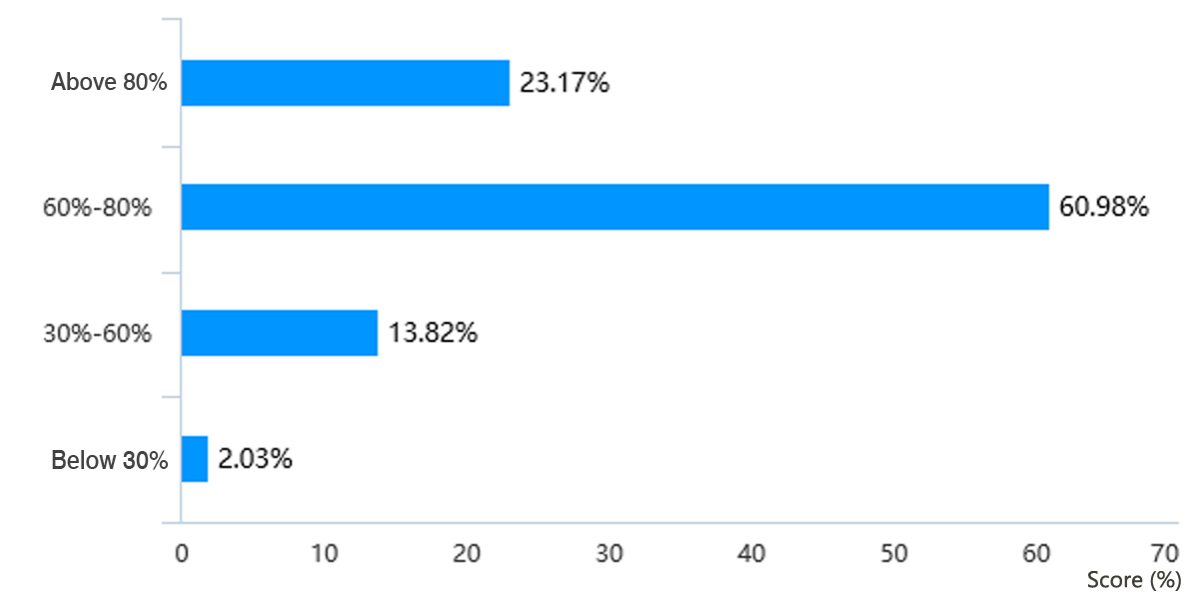
Scores showing the extent of students’ understanding and mastery of the theoretical knowledge of pathology through web-based learning.

**Figure 7:**
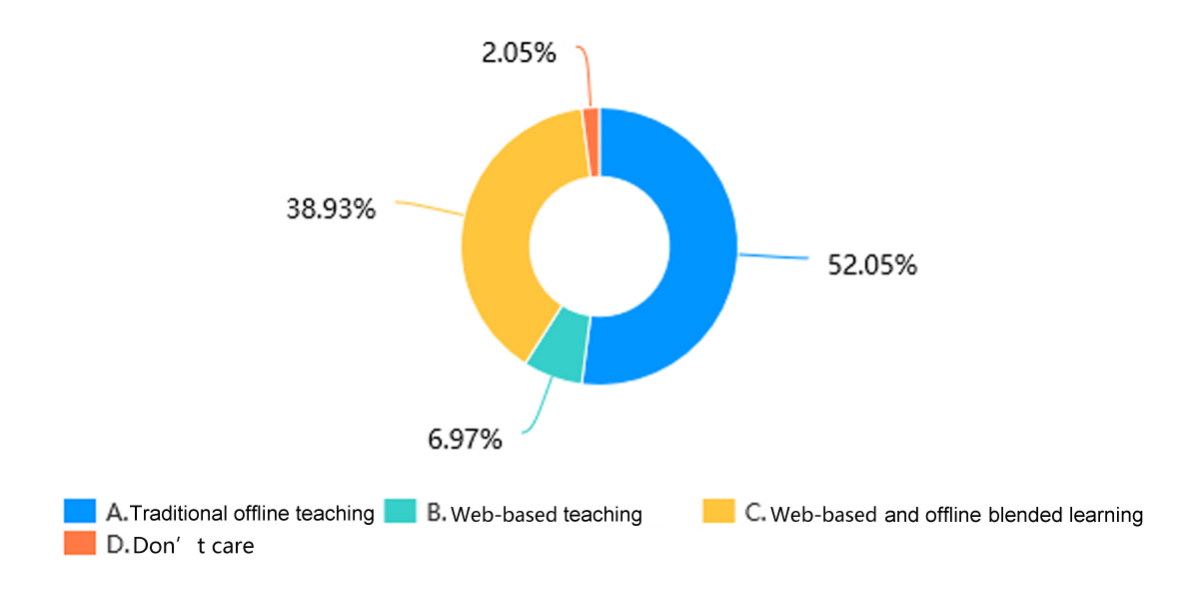
The proportions of students who chose different teaching methods of histology and embryology after the COVID-19 epidemic.

**Figure 8:**
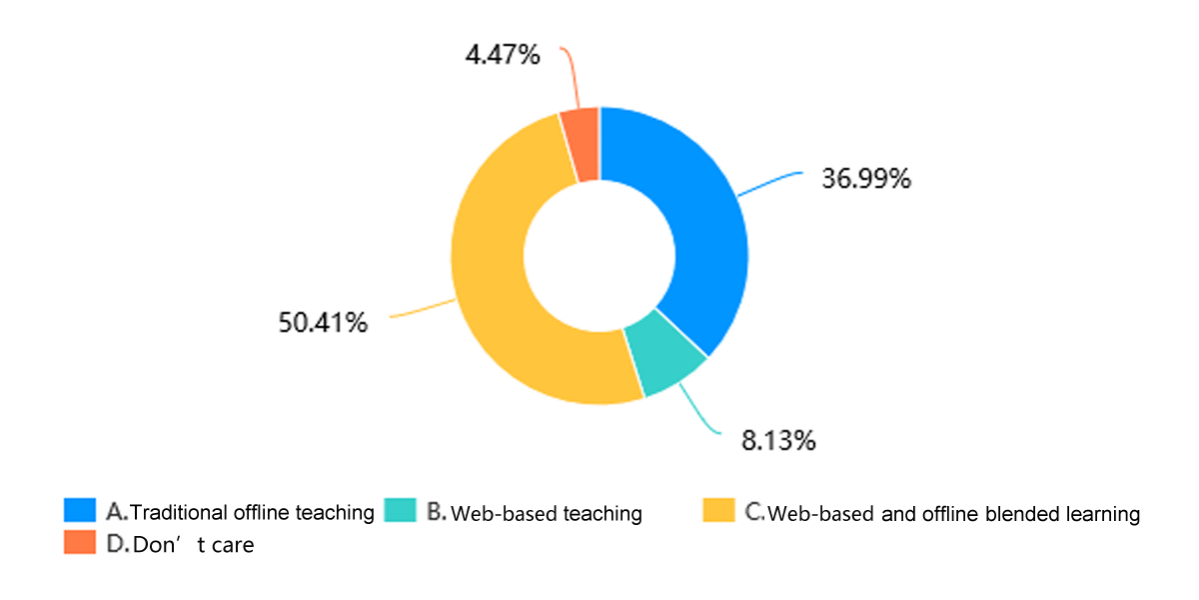
The proportions of students who chose different teaching methods of pathology after the COVID-19 epidemic.

**Figure 9:**
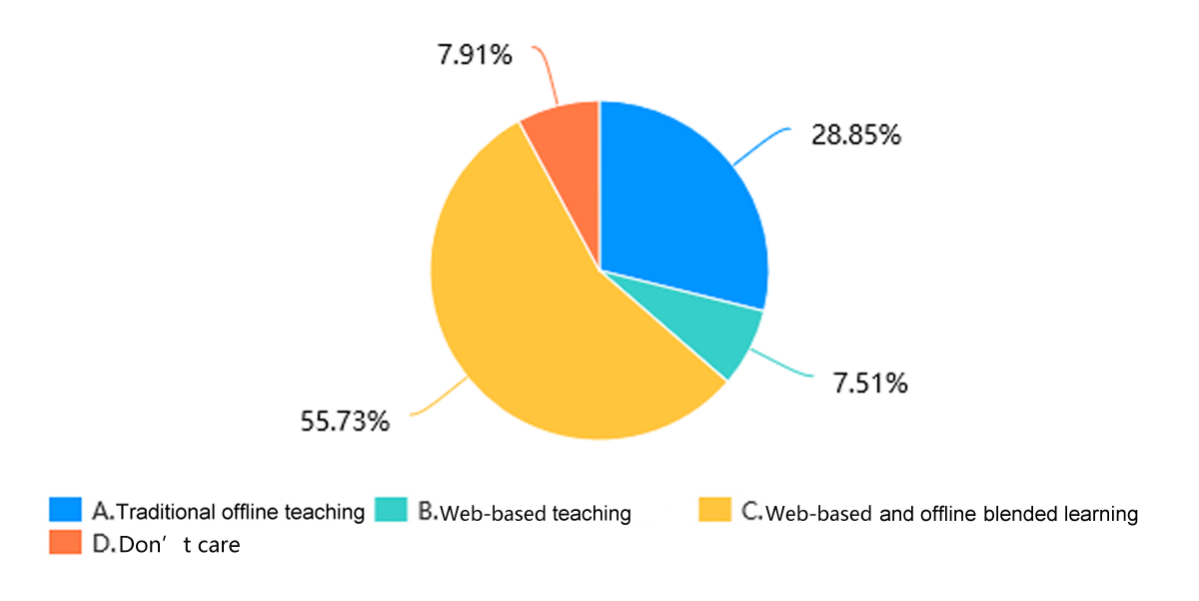
The proportions of students who would choose different teaching methods of histology and embryology in the future in the control group.

**Figure 10:**
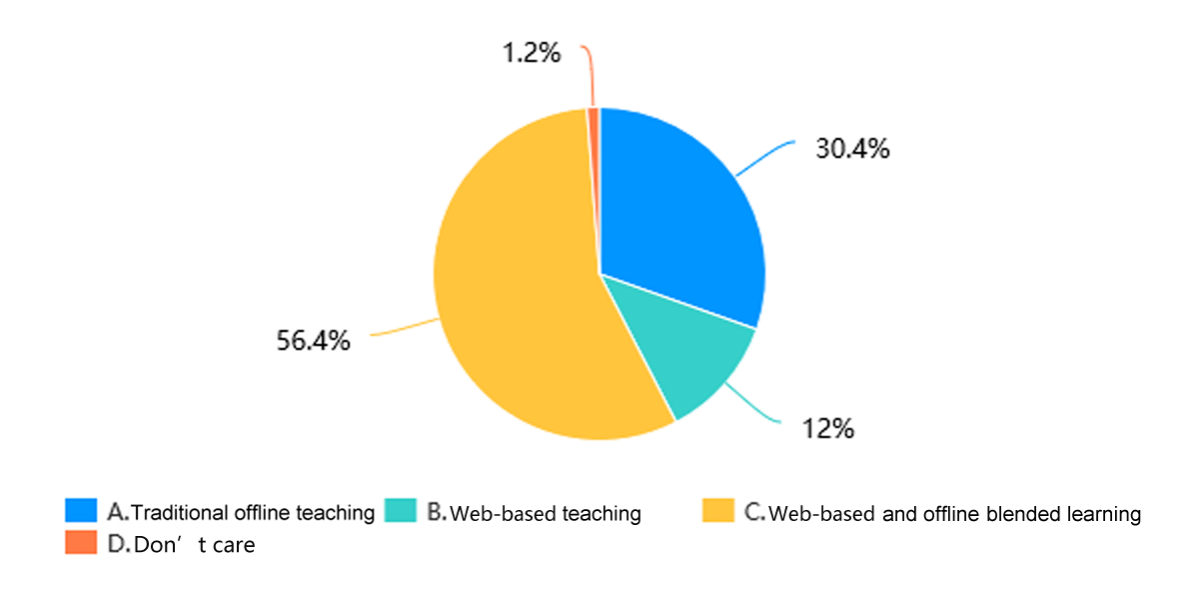
The proportions of students who would choose different teaching methods of pathology in the future in the control group.

Through web-based learning, more than 82.4% (201/244) of students in the Histology and Embryology course (Figure 5) and more than 84.1% (207/246) of students in the Pathology course (Figure 6) achieved a score of more than 60% in the course by gaining theoretical knowledge. 17/244 students (7.0%) in the Histology and Embryology course (Figure 7) and 20/246 students (8.1%) in the Pathology course (Figure 8) with strong self-learning ability stated they would opt for web-based learning in the future. Of the 246 students in the Pathology course who answered the survey, 91 (37.0%) wished to return to offline teaching, and 124 (50.4%) (Figure 8) preferred web-based and offline blended learning. Of the 244 students in the Histology and Embryology course who answered the questionnaire, 127 (52.1%) (Figure 7) wished to return to offline classroom teaching after the epidemic; meanwhile, in the control group, 19/253 (7.5%) students in the Histology and Embryology course (Figure 9) and 30/250 (12%) students in the Pathology course (Figure 10) opted for web-based learning in the future, while 141/253 (55.7%) students in the Histology and Embryology course (Figure 9) and 141/250 (56.4%) students in the Pathology course (Figure 10) preferred web-based and offline blended learning.

**Figure 11:**
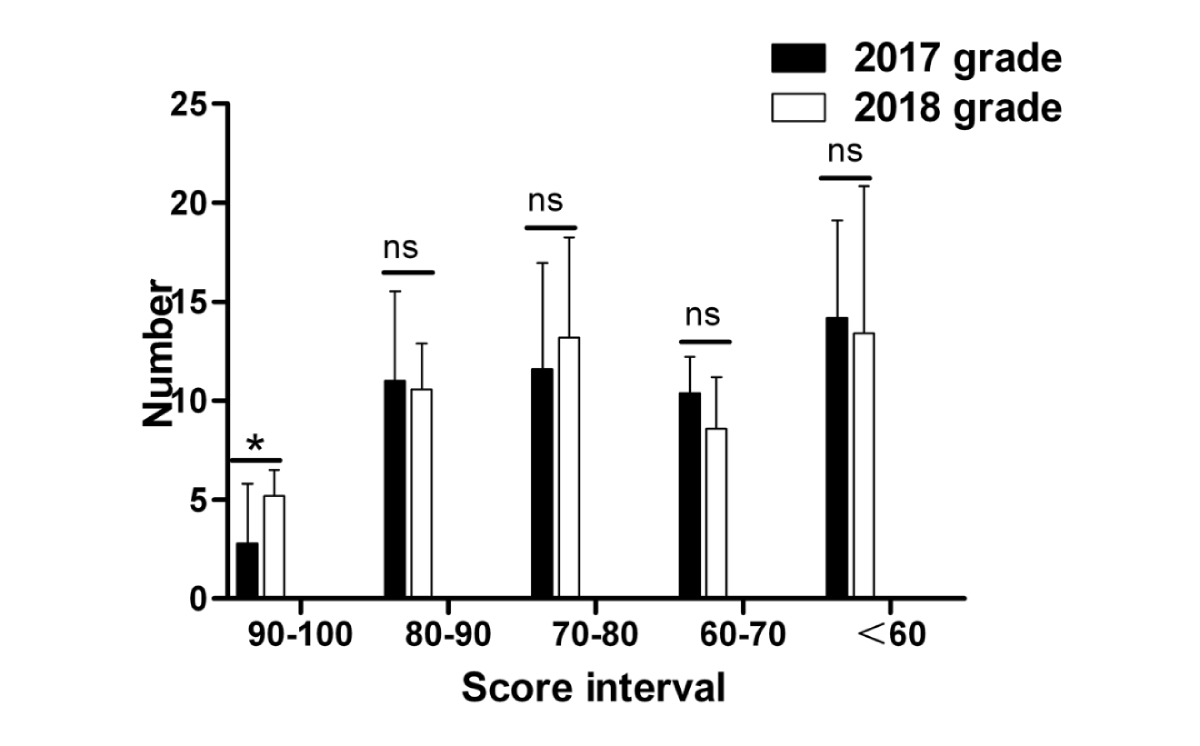
Statistical differences in the different score intervals in the final written pathology examination results. *: statistical significance; ns: no statistically significant difference.

**Figure 12:**
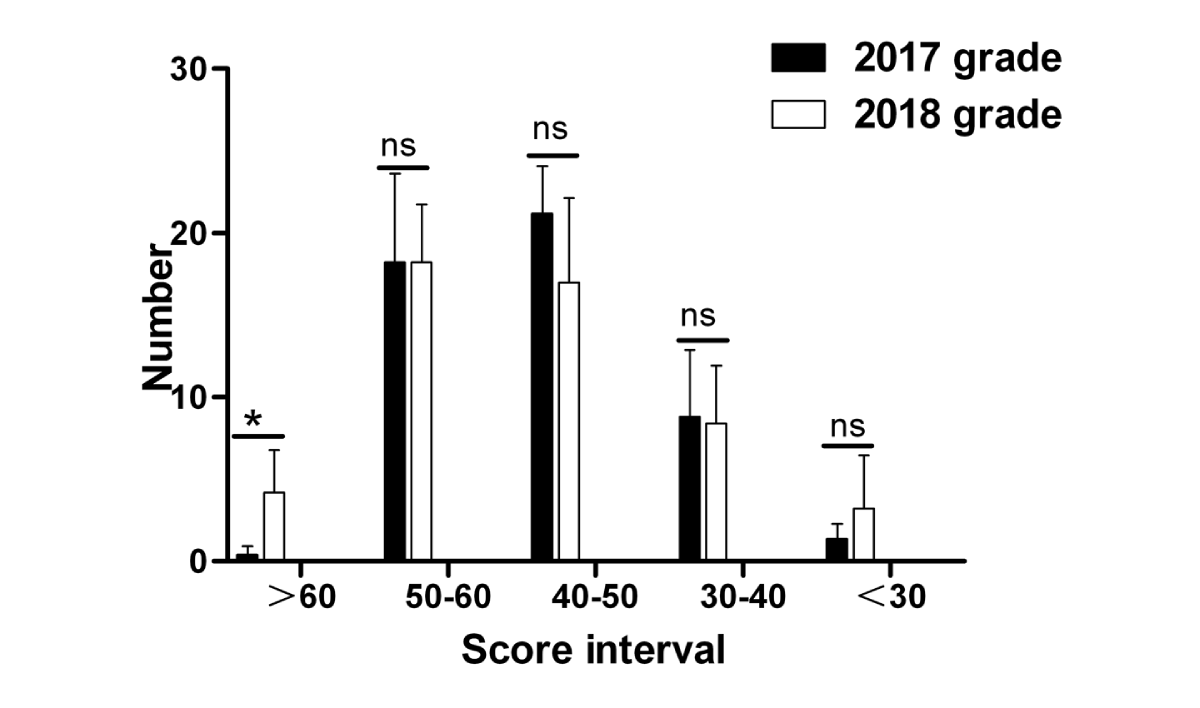
Statistical differences in the different score intervals for objective scores in the final written pathology examination results. *: statistical significance; ns: no statistically significant difference.

**Figure 13:**
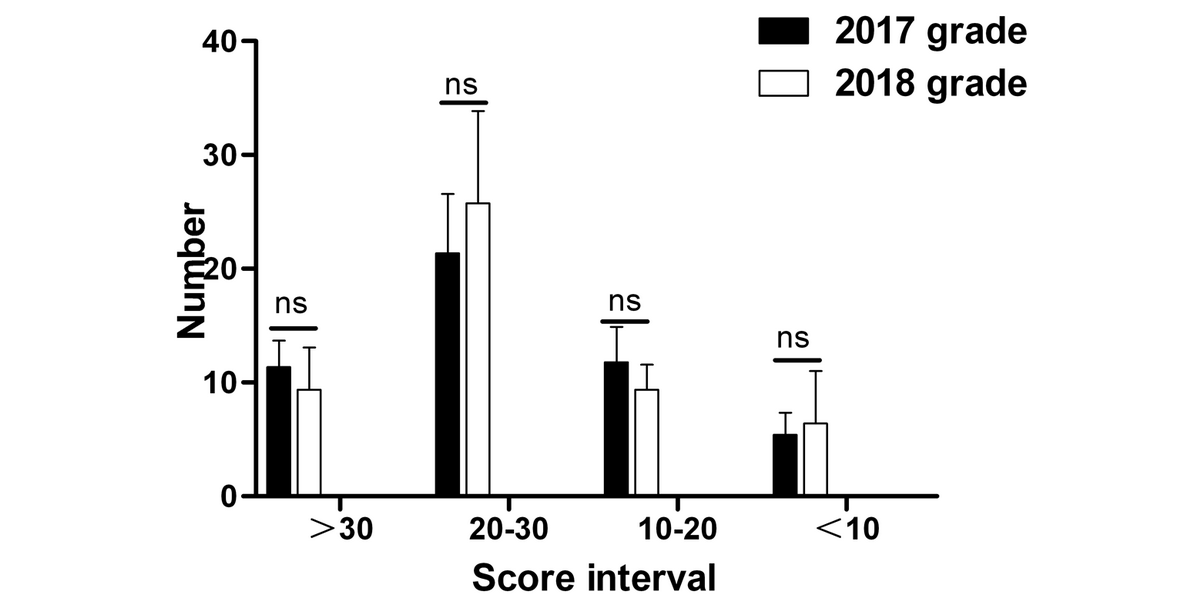
Statistical differences in the different score intervals for subjective scores in the final written pathology examination results. *: statistical significance; ns: no statistically significant difference.

A comparison of the final written examination scores of students in Grade 2018 with those in Grade 2017 showed that significantly more students in Grade 2018 scored between 90 and 100 than those in Grade 2017 (*P*=.02), while a few others obtained scores of 80-90, 70-80, 60-70, and <60; the number of students in Grade 2018 showed no significant differences from that of students in Grade 2017 (Figure 11). In terms of objective scores, the number of students who scored >60 in Grade 2018 was significantly higher than that in Grade 2017 (*P*=.045); meanwhile, for other scores, including 50-60, 40-50, 30-40, and <30, no significant difference was observed between Grade 2018 and Grade 2017 (Figure 12). Moreover, for the subjective questions, no significant difference was observed in any grade (Figure 13).

## Discussion

### Principal Results

Our teaching team emphasized “internet + education” and adopted a “Seven-in-One” teaching mode of videos, materials, chapter tests, interaction, homework, live broadcasts, and case analysis/discussion. We adopted six web-based applications, namely the Xuexi Tong platform, Tencent Live Conference, Chinese University MOOC/school MOOC, a medical morphology digital teaching platform, WeChat/QQ, and Questionnaire Star, to guide our web-based teaching. The teaching of morphological courses with a series of education methods ensured that the web-based teaching was not inferior to traditional offline teaching.

The teaching team explored a new teaching mode wherein it could attract the students’ attention and mobilize their enthusiasm for learning inside and outside the classroom. Teachers changed from knowledge imparters to instructors to help students learn, and they turned the “teaching-centered” notion into the “learning-centered” notion. Case analysis is an inquiry-based approach that can prompt students to actively engage in knowledge construction and develop competencies across multiple contexts [15,16]; it is required in the final written examination, and it is also a very good material to develop problem-based learning or Clinical Pathology Conference teaching. Our training mode of case analysis can cultivate clinical thinking ability in students. Considering the expression “A picture is worth a thousand words,” the training of writing mind maps can strengthen students’ understanding and memory of knowledge, improve their learning efficiency, cultivate divergent thinking, and encourage students to build a complete knowledge system and to integrate knowledge.

The teaching mode of unilateral instillation of knowledge through video has been changed, and the flipped classroom teaching approach was applied to strengthen the students’ interactions and to improve their “hands-on” experience. Hew and Lo’s study [17] indicated that more students favored the flipped classroom approach over traditional classroom teaching. Many of the in-class activities. such as small-group discussions, promoted students’ interactions with their peers in flipped classes. Teachers also felt that they had a greater opportunity to provide more feedback during in-class sessions. In addition, there were greater opportunities for students to apply their knowledge [18].

The experimental courses of Histology and Embryology and Pathology belong to the category of morphology, and both require microscopic observation of tissue sections. Virtual microscopy has advantages in learning histology and pathology; it enables users to view slides almost anytime or anywhere, produce annotations that enhance student learning, and integrate slides into other digital resources [19]. Through screen-to-screen experiment teaching, the classroom teaching content is consolidated and the theoretical course understanding is deepened.

Organizing a drawing competition not only consolidates students’ learning and understanding of human morphology but also improves their mastery and integration of knowledge of anatomy, histology and embryology, and pathology. This is more conducive for students to accept and master the surgical professional theories and their related skills.

Because COVID-19 is a “living teaching material,” the teachers simply used the opportunity of the theme activity to carry out a character-education movement of “patriotism, love for mankind, and love for medicine.” Many students who participated believed that in the face of the COVID-19 pandemic, it is necessary not only to solve problems using scientific knowledge but also to hone their minds and skills to become capable and responsible medical workers. All students said that they would take initiative to come forward when the country encounters difficulties in the future. This activity improved the students’ literary expression ability and thinking ability, and it also cultivated the students’ medical and humanistic qualities.

The data from the Questionnaire Star survey showed that the overall performance of web-based teaching remained good; however, 127/244 (52.1%) students in the Histology and Embryology course preferred to return to offline classroom teaching after the epidemic. According to our understanding of students undergoing web-based learning of theoretical knowledge, the reason for this finding is that the students experienced a strong desire for practical operations, such as making tissue slices and other hands-on experiments, which can only be realized by offline teaching. Therefore, students opted to go back to the classroom or chose web-based and offline blended learning.

We found that the final written examination results of the web-based learning students were not inferior to those who experienced traditional offline learning. There were more high-scoring students (90-100) in the Grade 2018 web-based teaching classes than in the Grade 2017 offline learning classes (*P*=.02). These results suggest that for those students with good ability to self-study, the web has become more conducive to learn and self-study, to summarize and expand, and finally to improve the corresponding results.

### Limitations

First, web-based teaching requires students to stare at the computer, mobile phone, or iPad for a long time; therefore, it is difficult to concentrate. There is a lack of school atmosphere; thus, students lacked interest in peer feedback [20]. Second, as web-based teaching was implemented throughout the country, the network jammed at times, and a few students had insufficient hardware at home and could only learn through the 4G network of mobile phones with insufficient traffic, restricting the implementation of web-based teaching to a certain extent. Third, the formative evaluations may have been affected by academic dishonesty and thus failed to objectively reflect the students’ scores [21]. In addition, this study was conducted during the emergency situation of the epidemic, and we lacked a suitable randomized control group. Moreover, the Questionnaire Star survey only focused on the perception and satisfaction of the network; therefore, longer-term research is warranted to evaluate the medium-term and long-term impact. Finally, this research lacked a rigorous sampling process, and all students in the classes of the experimental group and control group were included in our research.

### Conclusions

In short, the web-based teaching of histology and embryology and pathology in one semester demonstrated good performance and achieved the purpose of teaching with “suspension of classes without suspending the school.” This new teaching mode not only successfully satisfied the urgent needs of students to acquire knowledge during the epidemic period but also provided a practical foundation for blended learning in the future.

Web-based teaching has the advantage of being more flexible in enabling students to choose when and where to study. Under the new teaching mode that was established, the course content of web-based teaching showed no shrinkage, and the requirements for students were not reduced. The web-based teaching was simply the Level I response to “a public health emergency of international concern over the global outbreak of novel coronavirus” [22], and most universities will continue to conduct face-to-face learning [23]. Therefore, for the teacher, web-based teaching is a challenge as well as an opportunity. Moreover, teachers can take advantage of this opportunity to master relevant skills and methods by constant exploration and practice. The COVID-19 epidemic will have a profound impact on education for the foreseeable future [24].

Our teachers are the education reformers and the main force of development. We are not only teachers but also learners, researchers, and innovators. Web-based teaching and distance education are growing parts of medical education [25]. However, one of the problems in evaluating web-based teaching is the lack of a proper evaluation tool [26]. Therefore, we need to further consider this evaluation in future studies. Additionally, the main task of teacher training and development is to organically combine teacher learning techniques by using timely techniques to explore new education models that combine web-based and offline learning [8] and to use technology as an important carrier for the development of education.

Therefore, after the COVID-19 epidemic, the combination of web-based and offline teaching methods, which is a new trend, would be advocated. Meanwhile, teachers should change their teaching concepts, establish “internet + education” thinking modes, learn to share and use big data, and better meet and serve the learning needs of their students.

## Appendix

Multimedia Appendix 1 In the flipped classroom, a student used the tencent conference for presentation.

## Abbreviations

MOOC: massive open online course
